# Fluid resuscitation in haemorrhagic shock in combat casualties

**DOI:** 10.1186/s40696-017-0030-2

**Published:** 2017-01-17

**Authors:** Parli R. Ravi, Bipin Puri

**Affiliations:** 1 5 AFH, Rowriah Jorhat, 785005 India; 2 Office of DGAFMS, M Block, New Delhi, 110001 India

**Keywords:** Fluid resuscitation, Warm fresh whole blood, Coagulopathy, Damage control resuscitation

## Abstract

This brief update reviews the recent literature available on fluid resuscitation from hemorrhagic shock and considers the applicability of this evidence for use in resuscitation of combat casualties in the combat casualty care (CCC) environment. A number of changes need to be incorporated in the CCC guidelines: (1) dried plasma (DP) is added as an option when other blood components or whole blood are not available; (2) the wording is clarified to emphasize that Hetastarch is a less desirable option than whole blood, blood components, or DP and should be used only when these preferred options are not available; (3) the use of blood products in certain tactical field care settings where this option might be feasible (FSC, GH) is discussed; (4) 1:1:1 damage control resuscitation (DCR) with plasma: packed red blood cells (PRBC): platelets is preferred to 1:1 DCR with plasma: PRBC when platelets are available; and (5) the 30-min wait between increments of resuscitation fluid administered to achieve clinical improvement or target blood pressure has been eliminated. Also included is an order of precedence for resuscitation fluid options. There should be an emphasis on hypotensive resuscitation in order to minimize (1) interference with the body’s hemostatic response and (2) the risk of complications of over resuscitation. Hetastarch is retained as the preferred option over crystalloids when blood products are not available because of its smaller volume and the potential for long evacuations in the military setting.

## Background

About 25% of battle casualties are ‘potentially salvageable’ [1]. The single major cause of death over 6 h in this group is haemorrhage either as a primary insult or secondary to trauma induced coagulopathy. Fluid resuscitation of the right type and in the right dose reduces mortality and morbidity [2]. The concept of fluid resuscitation for haemorrhagic shock has changed from large volume crystalloid resuscitation, to small volume colloid resuscitation and use of blood or blood products at the earliest—even as a primary resuscitative fluid and early use of anti-fibrinolytic such as tranaxemic acid all of which form a part of damage control resuscitation [3]. Damage control resuscitation (DCR) comprises of hypotensive resuscitation and haemostatic resuscitation which aims to achieve the following:

- A restricted increase in intravascular volume targeting a systolic blood pressure (SBP) up to 90 mmHg (improvement of mental status or a weakly palpable radial pulse as a surrogate marker) so as to minimizes adverse effects of edema and prevent dilutional coagulopathy caused by large volume crystalloid resuscitation, reduce the incidence of a re-bleed due to clot dislodgement (which increasingly occurs over systolic blood pressures of 90 mm Hg) while maintaining perfusion to vital organs
- Enhance the body’s ability to form and retain a clot at bleeding sites
- Optimize oxygen carrying capacity

This commentary reviews the available fluids used in resuscitation and correct recommendations in vogue in management of combat casualties.

## Fluids used in resuscitation

### Crystalloids

Crystalloids in a ratio of 3:1 (3 ml of crystalloid for every ml of shed blood) are used commonly as the first line of fluid in haemmorghic shock. Crystalloids however, have no role in DCR as short term increase in intravascular volume are negated by risk of pulmonary oedema, displacement of forming clots at sites of vascular injury, abdominal compartment syndrome, acidosis, worsening of cerebral oedema, and dilutional coagulopathy [4]. In fact there is Level B clinical evidence that large volume crystalloid resuscitation reduces survival [5]. If the only available fluid is crystalloid then, ringer lactate is preferred over NS because it does not produce the hyperchloremic acidosis and the concern of lactate in RL causing acidosis is some what unfounded [6]. Plasma-lyte A if available offers some benefit over RL [neutral ph of 7.4, osmolarity of 295 mosm/l and no calcium. RL is more acidic, slightly hypotonic and contains calcium which may cause blood to coagulate if used with packed red blood cells (PRBC) or whole blood]. Hypertonic saline dextran (HSD) and hypertonic saline (HS) improve hemodynamic and metabolic responses, modulate immune function and reduce brain oedema in a number of experimental injury models. However a cochrane study concluded that there is no evidence that hypertonic crystalloids are better than near isotonic crystalloids for fluid resuscitation in trauma [7].

### Colloids

Colloids [Gelatins, various Hydroxy ethyl starches (HES) and albumin] are more effective than crystalloids for expanding the plasma volume because they contain large, poorly diffusible solute molecules that create an osmotic pressure to keep water in the vascular space with less extravasation of fluid into the lung than RL with a resulting improvement in oxygenation [8]. The smaller volume of colloids required for an equivalent and extended expansion of the intravascular space as compared to crystalloids offers a logistical advantage. However their propensity to cause renal damage is still controversial with some studies reporting no renal damage [7] while others did [9]. The FIRST trial reported that hydroxyl ethyl starch improves the renal function and lactate clearance when used for resuscitation in patients of penetrating trauma [9]. Additionally high molecular weight HES inhibits platelet function with the exception of Hextend (HES 130/0.75 in balanced electrolyte solution) which has a platelet stimulating effect which may be partially due to its solvent containing calcium chloride dehydrate [10]. There is Level B evidence that Hextend used in two aliquots of 500 ml each to supplement fluid resuscitation in trauma patients is safe and does not result in a coagulopathy [11–13]. However, the FDA issued a safety communication on HESs (Hespan, Hextend, and Voluven) in November 2013. The warning noted an increased risk in mortality and renal replacement therapy associated with the use of these products as used to treat critically ill patients [14]. However this communication did not mention the use of these products in the prehospital resuscitation of trauma patients, nor did it address the known increase in mortality and fluid overload complications resulting from the alternative use of large volume crystalloids in such patients Use of albumin for resuscitation in patients with associated traumatic brain injury (TBI) resulted in increased mortality [15]. This fact precludes its use in combat casualty care as haemorrhagic shock and TBI often co-exist.

### Blood and blood products

Resuscitation with whole blood, component therapy and plasma in that order best fulfil the criteria of DCR as they contribute to haemostasis in varying degrees while increasing the intravascular volume. There is some debate on the ability of Warm fresh whole blood and stored whole blood in correcting coagulopathies. Certain authors advocate fresh warm whole blood (FWWB) to more efficient than component therapy or stored whole blood in correcting coagulopathy as it has fresher RBC and better functioning and concentration of platelets and plasma [16]. The amount of fresh warm whole blood transfused is independently associated with improved 48-h and 30-day survival and the amount of stored red blood cells transfused is independently associated with decreased 48-h and 30-day survival for patients with traumatic injuries that require massive transfusion [17]. However few recent studies and reports have shown the efficacy of stored whole blood to be as good as FWWB [18, 19]. The use of FWWB in combat casualty care may be warranted stored whole blood or PRBC at the level of the field surgical centre (FSC) since the supply of the latter is likely to be small and component therapy is unavailable. Establishment of walking blood banks at FSC level where FWWB can be obtained stored and transfused is desirable. FWWB carries the risk of transfusion transmitted diseases which can be minimized by donor screening surveys, rapid screening tests of donated products for HIV, HCV and Hepatitis B, and pre-donation screening of potential donors (when possible) for routinely tested TTD’s. In addition all military personnel should be tested for HIV and immunized against Hepatitis B prior to deployment. Table 1 depicts the combat Casualty Care recommendations for the collection, treatment, and storage of FWWB.

**Table 1:** Guidelines for blood collection and storage in combat areas

Donation
Develop a pre-deployment roster of pedigreed donors (screened every 90 days)
ABO and Rh
Transmissible diseases
In emergency situations
Prefer previous and type O donor
Perform onsite ABO typing
Perform direct cross-match if possible
Treatment
Use approved blood recipient set(contains anticoagulant)
Fill until 650-mL bag is nearly full (approx 450 mL blood)
Draw cross-match and transmissible disease blood tubes
Submit to supporting lab even after use of blood
Storage
Keep fresh warm blood no longer than 24 h
If less than 8 h old, may be refrigerated for 3 weeks

Lyophilized (dried) plasma (DP) is a logistically attractive option for battlefield trauma care at the RAP as it can be stored under a wide range of temperature (2 to 35 °C) for 15–24 months. The two available brands currently available and are been used extensively in combat operations are LyoPlas and LyoPhil. DP offers the opportunity for both volume replacement and replacement of lost clotting factors and has a good safety record [20]. Thirty-eight percentage of combat casualties who require a transfusion have a coagulopathy [21] which increases mortality by three to sixfold [22] and plasma administration reduces the coagulopathy. The German DP product (LyoPlas) is a quarantined, single-donor product. When stored at room temperatures for 24 months, the individual coagulation factors retain 75–100% of their activity. LyoPlas also enables rapid treatment of coagulopathies without the need for complex logistics or thawing. Over 230,000 units have been transfused to date with no reports of major adverse complications to include viral transmission. The frequency of transfusion reactions approximates that of FFP. LyoPlas is type specific; type AB can be used if the recipient’s blood type is unknown. The Israeli Defence Force (IDF) has implemented a program to provide DP at the point of injury. The IDF program selected the German LyoPlas product, and it has now been used at the point of injury [20]. Presently this product is not available in India. All efforts should be made to ensure the availability of this wonderful resuscitative option for the Armed Forces. Thus the preferred fluids for DCR in haemorrhagic shock in order of preference are as under in order of preference:

1. Whole blood
2. 1:1:1 plasma, RBCs, and platelets
3. 1:1 plasma and RBCs
4. Reconstituted DP (not available presently), liquid or thawed plasma, alone or RBCs alone
5. Hextend
6. RL or Plasma-Lyte

Combat casualties are initially managed in an austere environment and choice of fluid will very often be confounded by logistical issues. Guidelines for resuscitation of combat casualties at various echelons First aid post and forward surgical centre (FAP and FSC) is given in Table 2. None the less all efforts should be made to ensure availability of blood, blood products and lyophilized plasma as far forward as possible to ensure best possible care to combat casualties. Early use of tourniquets, active and passive warming devices and early use of tranaxemic acid [22] when indicated play an important role in salvaging combat casualties suffering from haemorrhagic shock.

**Table 2:** Guidelines for fluid resuscitation at various field echleons

Fluid resuscitation at level of Combat Zone
Assess for hemorrhagic shock; altered mental status (in the absence of head injury) and weak or absent peripheral (radial)pulses are the best field indicators of shock
a. If not in shock:
No IV fluids necessary
PO fluids permissible if conscious and can swallow
b. If in shock:
Hextend, 500 mL IV bolus
Repeat once after 30 min if still in shock
No more than 1000 mL of Hextend
c. Continued efforts to resuscitate must be weighed against logistical and tactical considerations and the risk of incurring further casualties
d. If a casualty with an altered mental status due to suspected TBI has a weak or absent peripheral pulse, resuscitate as necessary to maintain a palpable radial pulse
Fluid resuscitation at First Aid post
Reassess for hemorrhagic shock (altered mental status in the absence of brain injury and/or change in pulse character.) Maintain target systolic BP 80–90 mmHg
a. If not in shock:
No IV fluids necessary
PO fluids permissible if conscious and can swallow
b. If in shock and blood products are not available:
Resuscitate with Dried Plasma (DP) or if not available
Simultaneously give 1gm of tranaxemic acid in 100 ml saline
Hextend 500 mL IV bolus
Repeat after 30 min if still in shock
Continue resuscitation with Hextend or crystalloid solution as needed to maintain target
BP or clinical improvement
c. If in shock and blood products are available under an approved command or theater protocol:
Resuscitate with whole blood preferably FWWB. Continue resuscitation as needed to maintain target BP or clinical improvement
d. If a casualty with an altered mental status due to suspected TBI has a weak or absent peripheral pulse, resuscitate as necessary to maintain a palpable radial pulse. If BP monitoring is available, maintain target systolic BP of at least 90 mmHg
Fluid resuscitation at Field Surgical Centre
a. The resuscitation fluids of choice for casualties in hemorrhagic shock, listed from most to least preferred, are: whole blood*; plasma, RBCs and platelets in 1:1:1 ratio*; plasma and RBCs in 1:1 ratio; plasma or RBCs alone; Hextend; and crystalloid (lactated Ringer’s or Plasma-Lyte A)
b. Assess for hemorrhagic shock (altered mental status in the absence of brain injury and/or weak or absent radial pulse)
1. If not in shock:
No IV fluids are immediately necessary
Fluids by mouth are permissible if the casualty is conscious and can swallow
2. If in shock and blood products are available under an approved blood product administration protocol:
Resuscitate with whole blood*, or, if not available
Plasma, RBCs, and platelets in a 1:1:1 ratio*, or, if not available
Plasma and RBCs in 1:1 ratio, or, if not available
Reconstituted dried plasma, liquid plasma or thawed plasma alone or RBCs alone
Reassess the casualty after each unit. Continue resuscitation until a palpable radialpulse,improved mental status or systolic BP of 80–90 mmHg is present
3. If in shock and blood products are not available under an approved combat theater blood product administration protocol due to tactical or logistical constraints:
Resuscitate with Hextend, or if not available
Lactated Ringer’s or Plasma-Lyte A
Reassess the casualty after each 500 mL IV bolus;
Continue resuscitation until a palpable radial pulse, improved mental status, or systolic
BP of 80–90 mmHg is present
Discontinue fluid administration when one or more of the above end points has been achieved
4. Ongoing resuscitation to continue along with damage control surgery (DCS)
5. At any given time all possibilities of MEDEVAC should be considered

## Conclusion

Fluid resuscitation strategies for combat casualties need to be urgently refined to keep pace with current recommendation. Large volume crystalloid resuscitation which is still in vogue in the most situations in combat need to be replaced with low volume colloid resuscitation and one of plasma or FWWB as forward in the field as possible to satisfy the arms of DCR namely the hypotensive resuscitation and haemostatic concerns. There should be a policy to outline planning parameters for blood supply to Forward medical echelons. The policy should specify zone wise responsibilities at various levels for ensuring availability of blood and blood products including collection, storage and transportation to meet operational requirements.

## References

[1] Eastridge BJ et al. Death on battlefield (2001-2011):implications for the future of combat casualty care. Trauma Acute care surg.2012;73(6 Suppl5): S431-7. 10.1097/TA.0b013e3182755dcc 23192066

[2] Bellamy RF,1995 combat trauma over view.pp 1- 42 in text book of military medicine eds Washington DC:TMM publications.

[3] Rt Gerhardt (2013). Remote damage control resuscitation and the Solstrand Conference: defining the need, the language, and a way forward. Transfusion..

[4] Rizoli SB (2003). Crystalloids and colloids in trauma resuscitation: a brief overview of the current debate. J Trauma.

[5] Bickell WH, Wall MJ, Pepe PE (1994). Immediate versus delayed fluid resuscitation for hypotensive patients with penetrating torso injuries. N Engl JMed..

[6] Schreiber M (2011). The use of normal saline for resuscitation in trauma. J Trauma.

[7] Bunn F,Roberts I, Tasker R et al. hypertonic versus near isotonic crystalloid for fluid resuscitation in critically ill patients Cochrane data bases yst Rev-2004;3 CD002045. 10.1002/14651858.CD002045.pub2 PMC7017932 15266460

[8] .Pape A, Kutschker S, Kertscho H, et al. The choice of the intravenous fluid influences the tolerance of acute normovolemic anemia in anesthetized domestic pigs. Crit Care.2012. 10.1186/cc11324 PMC3681398 22546374

[9] Ryan M, Ogilvie M, Pereira B (2012). Effect of hetastarch bolus in trauma patients requiring emergency surgery. J Spec Oper Med..

[10] .Lissauer M, Chi A, Kramer M, et al. Association of 6% hetastarch resuscitation with adverse outcomes in critically ill trauma patients. Am J Surg.2011. Epub ahead of print. 10.1016/j.amjsurg.2010.05.002 21600555

[11] MFM James, WL Michell et al. Resuscitation with hydroxyethyl starch improves renal function and lactate clearance in penetrating trauma in randomized controlled study: the FIRST trial (Fluids in resuscitation of severe trauma).BJA 2011;107(5): 693-702. 10.1093/bja/aer229 21857015

[12] The Effects of High Molecular Weight Hydroxyethyl Starch Solutions on Platelets. Deusch, Engelbert MD; Thaler, Ulrich MD; Kozek-Langenecker, Sibylle A. MD Anesthesia & Analgesia: September 2004 - Volume 99 - Issue 3 - pp 665-668. 10.1213/01.ANE.0000130349.99727.58 15333389

[13] Ogilvie M, Ryan M, Proctor K (2011). Hetastarch during initial resuscitation for trauma. J Trauma.

[14] FDA Safety Communication. Boxed warning on increased mortality and severe renal injury, and additional warning on risk of bleeding, for use of hydroxyethyl starch solutions in some settings.http://www.fda.gov/biologicsbloodvaccines/safetyavailability/ucm35827.htm.

[15] Myburgh J, Cooper DJ, Finfer S (2007). Saline or albumin for fluid resuscitation in patients with traumatic brain injury. N Engl J Med.

[16] Armand R, Hess JR (2003). Treating coagulopathy in trauma patients. Transfus Med Rev.

[17] Spinella PC. Warm fresh whole blood transfusion for severe hemorrhage: U.S. military and potential civilian applications. Crit Care Med. 2008 Jul; 36(7 Suppl):S340-5. 10.1097/CCM.0b013e31817e2ef9 18594261

[18] Spinella PC (2016). Whole blood for haemostatic resuscitation of major bleeding. Transfusion..

[19] Chen J et al. Limited resuscitation with fresh or stored whole blood corrects cardiovascular and metabolic function in a rat model of polytrauma and hemorrhage.Shock.2016 Sep 19. (Epub ahead of print). 10.1097/SHK.0000000000000748 27648698

[20] Glassberg E, Nadler R, Rasmussen TE (2013). Point-of-injury use of reconstituted freeze dried plasma as a resuscitative fluid: a special report for prehospital trauma care. J Trauma Acute Care Surg..

[21] Niles SE, McGlaughlin DF, Perkins JG (2008). Increased mortality associated with early coagulopathy of trauma in combat casualties. J Trauma.

[22] TranexamicAcid (TXA) in Tactical Combat Casualty Care. Guideline Revision Recommendation Committee on Tactical Combat Casualty Care 11 August 2011.

